# Sound-Action Symbolism

**DOI:** 10.3389/fpsyg.2021.718700

**Published:** 2021-09-14

**Authors:** Lari Vainio, Martti Vainio

**Affiliations:** ^1^Phonetics and Speech Synthesis Research Group, Department of Digital Humanities, University of Helsinki, Helsinki, Finland; ^2^Perception, Action, and Cognition Research Group, Department of Psychology and Logopedics, Faculty of Medicine, University of Helsinki, Helsinki, Finland

**Keywords:** sound symbolism, speech, action, grasping, prosody, gestures

## Abstract

Recent evidence has shown linkages between actions and segmental elements of speech. For instance, close-front vowels are sound symbolically associated with the precision grip, and front vowels are associated with forward-directed limb movements. The current review article presents a variety of such sound-action effects and proposes that they compose a category of sound symbolism that is based on grounding a conceptual knowledge of a referent in articulatory and manual action representations. In addition, the article proposes that even some widely known sound symbolism phenomena such as *the sound-magnitude symbolism* can be partially based on similar sensorimotor grounding. It is also discussed that meaning of suprasegmental speech elements in many instances is similarly grounded in body actions. Sound symbolism, prosody, and body gestures might originate from the same embodied mechanisms that enable a vivid and iconic expression of a meaning of a referent to the recipient.

## Introduction

The core elements of language have evolved nearly exclusively in face-to-face interaction. Typically, in face-to-face communication signaling, a meaning of a referent to the interpreter occurs *via* verbal and non-verbal communication channels. Verbal signaling consists of spoken words, while non-verbal signaling can utilize oral (e.g., prosody and laughing) and non-oral (e.g., manual gestures, facial expressions, and body postures) forms. Unlike in non-verbal signaling, the relationship between a form of the verbal sign and its meaning has been considered to be essentially arbitrary (e.g., Hockett, 1963). This view highlights that there is nothing inherent, for example, in the word *dog* to indicate what it represents. In contrast to this view, the idea of a non-arbitrary relationship between the verbal sign and its meaning has a long history dating back to Plato’s Socratic dialogue *Cratylus*. For example, Peirce (1931) has emphasized that many linguistic signs do not comply with the rule of arbitrariness, but rather iconically represent the referent object, such as in onomatopoeia (e.g., *knock*, *ring*, and *bang*). Later studies have recognized a variety of consistent non-onomatopoeic associations between speech sounds and concepts in which the sound iconically represents some aspect of an object, such as its size or shape (see Lockwood and Dingemanse, 2015 for a review).

This review provides a new theoretical perspective on iconic properties of speech by emphasizing insights derived from views of embodied cognition. The view assumes that many concepts – in particular, those that have relevance to actions performed with body parts – are essentially grounded in action representations (Barsalou, 2008; Pulvermüller and Fadiga, 2010). This view is in line with the motor chauvinist perspective (Sperry, 1952; Wolpert et al., 2001), according to which cognitive and perceptual functions have evolved to support motor behavior and hence, to a great degree, still operate in integration with motor processes. This review paper emphasizes that some sound symbolism phenomena can be partially based on conceptual grounding in motor processes. In support of this notion, the paper presents several recently observed sound symbolism effects that are based on an association between speech sound and action (e.g., Vainio et al., 2013, 2015). As an example, front vowels are associated with forward-directed limb movements, and close-front vowels are associated with the precision grip.

The paper proposes that these sound-action symbolism effects are based on tight linkages between the motor processes of articulatory mouth movements and movements of other body parts – the hands in particular. This view holds that, for example, close-front vowels are sound symbolically associated with the precision grip because the meaning of this grip is represented within a motor network that integrates the motor program of the precision grasp with the motor program related to the articulatory gesture of a close-front vowel. In addition, the paper suggests that some commonly reported sound symbolism effects, such as *the sound-magnitude effect* (Sapir, 1929), which have not been traditionally explained in terms of embodied accounts of cognition, can be also based on conceptual grounding in articulatory and manual sensorimotor processes. The paper further discusses that sound symbolism elements of spoken language have, to some degree, a common embodied origin with gestural and prosodic elements of communication; they are similarly, to a great extent, oriented to communicating ideas in a relatively iconic manner and are also grounded in action representations. However, before getting into the motor perspectives regarding iconic communicative signs, we discuss some basic principles concerning sound symbolism.

## Sound-Meaning Iconicity

In addition to direct imitation of sounds as observed in onomatopoeia, many languages have an extensive word class of non-onomatopoeic ideophones (also known as “expressives” or “mimetics”) that iconically utilize aspects of the speech signal that go beyond the absolute imitation of sound in order to depict aspects of meaning (Childs, 1994; Mikone, 2001; Schultze-Berndt, 2001; Bodomo, 2006). Often these ideophones include segmental sounds or sound structures that, instead of imitating a sound, are used to express a physical movement, a perceptual property, an affective state, or an action. For example, in Japanese ideophones, the rotation is often represented in the combination of consonants “g”/“k” and “r” (e.g., *koro* means “a light object rolling”); relatively large mass is represented using a voiced initial consonant (i.e., *goro* means “a heavy object rolling”); while reduplicating of a word indicates that the event occurs repeatedly (i.e., *gorogoro* means “a heavy object rolling continuously”; Kita, 1997, 2001; Childs, 2001). Experimental evidence showing, for instance, that participants guess the correct meaning of ideophones of a language unknown to the participant at an above-chance level of accuracy (Iwasaki et al., 2007; Dingemanse et al., 2016) has supported the view that such non-onomatopoeic ideophones present non-arbitrary connections between a vocal sign and meaning.

In this review paper, the term “iconicity” is defined as “the resemblance-based mapping between aspects of form and meaning” (Dingemanse et al., 2015). Onomatopoeic ideophones show “direct iconicity” between the meaning and phonetic aspects of a word, as they include sound elements that imitate the sound of a referent (Masuda, 2007). Based on the same definition, non-onomatopoeic ideophones show “indirect iconicity” between meaning and phonetic aspects of a word, as they associate an impression of a specific sound element of a word to a meaning. The above mentioned observations of direct and indirect iconicity are often labeled under the banner of “sound symbolism,” although it is noteworthy that in many, if not most, cases of such linguistic iconicity, the relation between meaning and sound is not based on convention, and many of the sound symbolism phenomena might be rooted in other underlying properties of speech rather than *sound*, such as the articulatory configurations discussed below.

The existence of iconicity, of course, does not invalidate the premise that arbitrariness is a core property of language. Iconicity and arbitrariness coexist in language, and both have their own functions (Dingemanse et al., 2015). For example, iconicity facilitates language learning (Imai and Kita, 2014; Nielsen and Dingemanse, 2021) as well as the comprehension of communicative signs (Perniss and Vigliocco, 2014; Nielsen and Dingemanse, 2021) and makes communication more vivid (Lockwood and Dingemanse, 2015). However, iconic sounds cannot easily be used to express many, if not most, concepts. Importantly, form-meaning arbitrariness allows language to denote potentially limitless concepts and is particularly useful in conveying abstract meanings (Lupyan and Winter, 2018).

The two most commonly investigated sound-meaning phenomena representing indirect iconicity, both cross-linguistically and in laboratory experiments, are *the bouba-kiki effect* (also known as *the maluma-takete effect*) originally observed by KÖhler (1929), and *the sound-magnitude effect* originally observed by Sapir (1929). In *the bouba-kiki effect*, curved shapes are associated with vowels, which involve lip rounding and/or tongue backing/lowering (i.e., [u], [ɑ], and [o]) as well as with the sonorant and voiced bilabial consonants (e.g., [m], [n], and [l]). In contrast, more jagged shapes are associated with relatively high and front vowels (i.e., [i] and [e]) and the voiceless stop consonants (e.g., [t], [k], and [p]; Tarte and Barritt, 1971; Ramachandran and Hubbard, 2001; Maurer et al., 2006). This effect has been observed many languages, such as English (KÖhler, 1929; Winter and Perlman, 2021), Swahili (Davis, 1961), Himba (Bremner et al., 2013), and Tamil (Ramachandran and Hubbard, 2001). In *the sound-magnitude effect*, high and front vowels are typically associated with small objects/concepts, while low and back vowels are associated with large objects/concepts (Birch and Erickson, 1958; Thompson and Estes, 2011). Similarly to *the bouba-kiki effect*, the *sound-magnitude effect* has been found in many languages, such as English (Johnson, 1967), Korean (Kim, 1977), as well as several other languages (Gebels, 1969; Newmeyer, 1992).

Although *the bouba-kiki effect* and the *sound-magnitude effect* have drawn a great deal of attention from researchers, languages include a much wider variety of sound-meaning correspondences, as also seen in the research on ideophones discussed above. In fact, Blasi et al. (2016) showed that a considerable proportion of the basic words of the world’s languages convey non-arbitrary sound-meaning associations. Cross-linguistic research has identified systematic sound-to-meaning mappings in relation to a range of referents, such as brightness (Hirata et al., 2011), colors (Johansson et al., 2020), body parts (Blasi et al., 2016), and emotions (Adelman et al., 2018). These sound-meaning associations can be conceptualized, in general, to arise from associations between particular conceptual and/or perceptual properties of a referent and some quality of the speech production (e.g., their articulatory and/or acoustic features).

Most explanations of sound-meaning associations have emphasized the involvement of cross-modal mappings between two or more processing modalities (e.g., Ramachandran and Hubbard, 2001; Sidhu and Pexman, 2018). In fact, it is well known that processing in separate modalities can be inextricably linked to one another, such as between smell and taste (Stevenson and Oaten, 2010), vision and hearing (McGurk and MacDonald, 1976), vision and action (Franca et al., 2012), or mouth and hand movements (Salmelin and Sams, 2002). As such, sound-meaning associations may be seen as an outcome of the associative pairing of percepts – based, for instance, on their temporal co-occurrence – that are essentially processed in separate systems (Keough et al., 2019). As an example, nasal consonants may occur relatively frequently in the word referring to *nose* across world languages (Greenberg, 1978; Blasi et al., 2016) because producing nasal consonants resonates in the nasal cavity, implicitly associating the nose with the particular sound of nasal consonants (Urban, 2011). Following this logic, over time, these kinds of systematically occurring cross-modal associations have established themselves in the lexicons of spoken languages.

Regarding the *sound-magnitude effect*, the effect has been most commonly linked to cross-modal mappings between acoustic properties of specific vowels and small/large objects. This acoustic account highlights that closed vowels typically have higher fundamental frequency (*f*_0_) than open vowels across different languages (Whalen and Levitt, 1995) perhaps because the heightening tongue “pulls on the larynx, and thus increases the tension of the vocal cords” (Ohala and Eukel, 1987, p. 207). Consequently, they can be cross-modally associated with smaller objects because smaller things tend to resonate at higher frequencies (Ohala, 1984; Spence, 2011). For instance, small animals have small vocal apparatuses, resulting in the production of higher frequencies compared to larger animals (Ohala, 1994). In addition to *f*_0_, empirical evidence shows that the *sound-magnitude effect* is also linked with the formants *F1*, which reflects tongue lowering, and *F2*, which reflects tongue fronting (Fant, 1960), so that larger objects are linked with increased *F1* and decreased *F2* (Knoeferle et al., 2017; Vainio, 2021). Furthermore, Fitch (2000) has proposed that *F1* and *F2*, and their differential spacing (formant dispersion), which is tied to vocal tract length and body size and decreases from high-front vowels to low-front vowels, might be a better indicator of body size than pitch. In line with this, Ohala (1994) have mentioned that the *sound-magnitude effect* may also depend on formant dispersion. Hence, research has shown some evidence for supporting the acoustic account of the *sound-magnitude effect* showing that associating specific vowels with small/large sizes can be attributable to acoustic characteristics of these vowels.

Taken together so far, languages include a wide variety of non-arbitrary associations between vocal signs and meaning that are likely to be attributable to a variety of cross-modal processes. However, the mechanisms behind different sound symbolism phenomena are still largely under debate. For example, although it is indeed intuitive to assume that nasal consonants are associated with nose because they resonate in a nose, conclusive empirical evidence are lacking for this explanation. There is an ongoing debate about the mechanisms underlying even *the bouba-kiki* and *the sound-magnitude effects* (Sidhu and Pexman, 2018), which are the most heavily explored sound symbolism effects. In the upcoming sections, we report a number of sound symbolism phenomena that present indirect iconic associations between speech sounds and actions. Based on these findings, we present a novel category of sound symbolism (i.e., sound-action symbolism) and propose the mechanistic underpinnings of this phenomenon.

## Sound-Action Symbolism

Viewed from the perspective of the motor chauvinists (Sperry, 1952; Wolpert et al., 2001) or embodied cognition (Barsalou, 2008; Pulvermüller and Fadiga, 2010), introduced in greater detail in the “Embodiment of Concepts,” motor processes can be viewed to contribute to sound symbolism effects. One view regarding how vocal sounds might be associated with actions assumes that certain articulatory gestures mimic or mirror some attribute of an object, such as its size or shape. Thus, a particular vocal sound, which is a consequence of producing the mimicking articulatory gesture, becomes cross-modally connected to the percept of the object. For example, regarding *the bouba-kiki effect*, some researchers have proposed that rounded vowels are linked to round-edged shapes in *the bouba-kiki effect* because lip rounding mirrors the round shape of the object and provides a cross-modal association between the sensorimotor percept of producing the rounded vowels and the visual percept of round-edged shapes (Ramachandran and Hubbard, 2001; Maurer et al., 2006). Regarding *the sound-magnitude effect*, it has been proposed that in addition to the acoustic account of the effect, introduced in the “Sound-Meaning Iconicity,” the effect can be also based on the association between the size of a referent and the sensorimotor percept of producing front-close/back-open vowels (i.e., the articulatory account). This view assumes that, for example, the back-open vowels gesturally mimic largeness of the referent by enlarging the vocal cavity and the front-close vowels gesturally mimic smallness of the referent by reducing the vocal cavity (Sapir, 1929; Ramachandran and Hubbard, 2001). If this view is applied to sound symbolically associating perceived phonemes with different magnitudes, it could be assumed that hearing, for example, the vowel [i] results in simulating this vowel in the articulatory motor processes that are involved in producing this vowel by reducing a vocal cavity (D’Ausilio et al., 2009), which in turn leads to decoding this vowel as small.

In line with the view that *the bouba-kiki* and *the sound-magnitude effects* are linked to vowel articulation processes, research shows that both of these effects can be observed in vowel production. Firstly, it has been demonstrated that when participants are required to articulate the vowel [i] or [u] according to the size of the round or sharp shapes, the articulation of [i] is facilitated by sharp shapes while [u] is facilitated by round shapes (Vainio et al., 2017b). Correspondingly, the articulation of unround-closed-front vowels is facilitated by processing small spatial and temporal aspects of stimuli, while articulation of round, open, and back vowels is facilitated by processing large spatial and temporal aspects of stimuli (Vainio, 2021). This evidence suggests that articulatory processes are associated with *the bouba-kiki effect* as well as *the sound-magnitude effect*.

Given this, it should be however noticed that conclusive and causal evidence for the acoustic as well and articulatory account of *the sound-magnitude effect* as well as the *bouba-kiki effect* is still lacking. Sidhu and Pexman (2018) have provided a comprehensive review of potential sound symbolism mechanisms proposing that there is such wide variability of sound symbolism phenomena that many different sound symbolism effects can be based on different mechanisms, and correspondingly a specific sound symbolism effect can be based on more than one mechanism. As an example, although lip rounding might map rounded vowels with round-edged shapes in the *bouba-kiki effect*, it has been also shown that participants associate sine-wave versions of the pseudowords *maluma* and *taketa* with round and sharp-edged shapes, respectively (Silva and Bellini-Leite, 2020), suggesting that purely acoustic properties of these words can provide characteristics that enable linking them to particular shapes. Similarly, the acoustic and articulatory accounts of the *sound-magnitude effect* are not mutually exclusive, and it is possible that both of them are valid.

### Sound Action Symbolism and Body Movements

In addition to the above-mentioned sound symbolic interaction between articulation and processing shape and the magnitude elements of a percept, another way in which segmental elements of speech can be iconically associated with actions has been presented by Imai et al. (2008). In their study, the authors created several novel ideophonic verbs expressing different manners of walking along the dimensions of speed and heaviness of movement as well as size of steps. Sound-meaning associations in these novel verbs were based on real sound-meaning associations observed in Japanese ideophones. In addition, the researchers created two video clips for each novel ideophone, with a character walking in a manner that hypothetically either matched or did not match the iconic sound elements of the ideophone. It was found that children as young as 2years of age were able to detect a sound–action match between ideophones and actions.

In addition to whole-body actions, sound-meaning iconicity can also be linked to hand actions. Many ideophones are often accompanied by gestures imitating the action conveyed by the ideophone (Kunene, 1965). In line with this, it has been shown that co-expressive iconic gestures are frequently produced at the time of vocalizing mimetic words when speakers describe motion and action (Kita, 2001). Similarly, Japanese mimetics are almost always – significantly more frequently than normal verbs – synchronically accompanied by manual gestures (Kita, 1997). These studies suggest that semantic expression of sound symbolic words and manual gestures originates from the same expressive representational processes that are tightly anchored on manual motor system (Kita, 1997). In line with this view, Perlman et al. (2015) have correspondingly suggested that vocal iconicity can originate from the same process of expressing thoughts as iconic gestures.

### Grounding Sound Action Symbolism in Reaching and Grasping

Originally, Ramachandran and Hubbard (2001) took the idea of the linkage between hand actions and sound-meaning associations to a precise level by proposing that articulatory gestures for words such as *teeny* ([ti:ni]) and *petite* ([pəˈti:t]) may mimic a precision grip gesture made by the index finger and opposing thumb. This view holds that the sound-meaning association between the concept of *smallness* and particular phonemes (e.g., the front-close vowels and the voiceless stop consonants) is mediated by cross-domain interaction between manual motor and mouth motor processes. This view is in turn based on the relatively long-lived mouth-gesture theory (Wallace, 1881; Paget, 1944; Hewes, 1973), which assumes that people have an innate tendency to mirror their own as well as others’ manipulative and communicative hand movements with analogous movements of the lips, mouth, and the tongue. This tendency can be observed, for example, when a manual cutting action is accompanied by a synchronous jaw opening/closing movement, or when one tries to thread a needle, and one’s finger movements are often accompanied by tongue-thrusts, as noted already by Darwin (1872). The mouth-gesture account holds that, over time, some vocal signs have become systematically linked with elements of manual gestures *via* this inherited imitative tendency. More recent evidence have supported the view that mouth and hand movements are programmed – to some extent – by combined motor processes (e.g., Arbib, 2005; Gentilucci and Corballis, 2006; Vainio, 2019). For example, the single-cell recordings from macaque monkeys’ premotor area F5 (the ventral premotor cortical area 6) contains neurons that discharge when the monkey grasps the object with the mouth or hand (Rizzolatti et al., 1988), and when the monkey observes grasping actions regardless of whether the grasping is executed with the hand or mouth (Gallese et al., 1996). In sum, evidence suggests that certain mouth actions and grasp-related manual actions are programmed within an overlapping sensorimotor network, and as a consequence they mutually influence each other.

**Figure 1:**
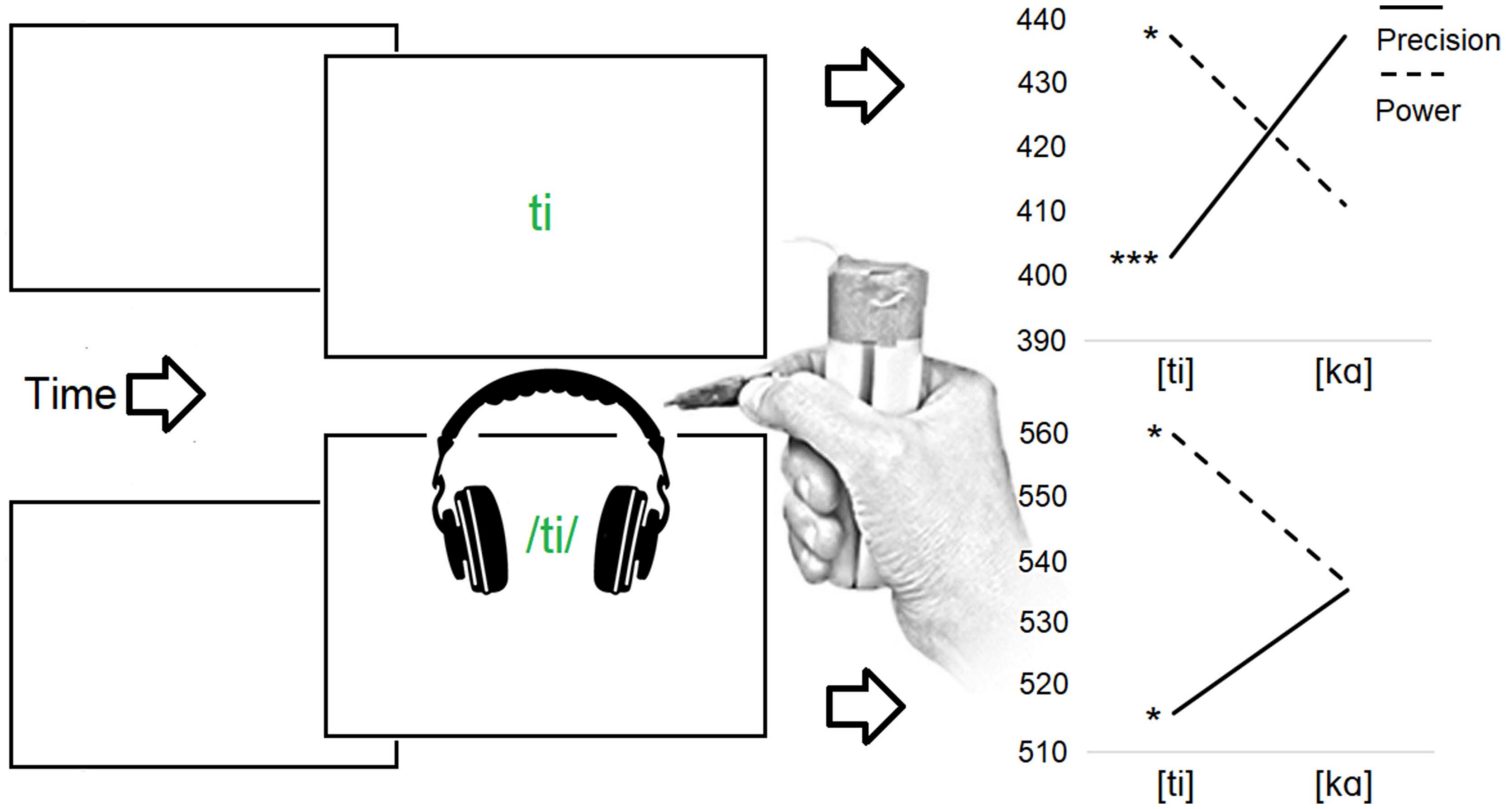
**(Above)** The reaction time (RT) effect for the study published by Vainio et al. (2013). In the experimental task, the participants were visually presented with a syllable (e.g., [ti] or [ka]). The participants had to respond by squeezing a precision or power grip device according to the color (green/blue) of the syllable while simultaneously pronouncing the syllable. Precision grip responses were performed particularly and rapidly with the syllable [ti], while power grip responses were performed particularly rapidly with the syllable [ka]. **(Below)** The RT effect for the study published by Vainio et al. (2014). In the experimental task, the participants were aurally presented with the syllable [ti] or [ka] at a high or low pitch. The participants had to perform either precision or power grip response based on the pitch cue. Precision grip responses were performed particularly rapidly with the syllable [ti], while power grip responses were performed particularly rapidly with the syllable [ka]. Asterisks indicate statistically significant differences (^***^p<0.001; ^*^p<0.05).

Importantly, our research has supported the proposal that the precision grip action is indeed associated with front-close vowels and voiceless stop consonants. In the paradigm originally reported by Vainio et al. (2013) (see also Tiainen et al., 2017a; Vainio et al., 2018), the participants were required to perform a dual action by simultaneously pronouncing a vowel/consonant and performing a grip response. The participants were visually presented with a vowel (e.g., [i] or [ɑ]) or a consonant (e.g., [t] or [k]) colored green or blue while they were holding a precision and power grip response device in their hand. They were asked to pronounce the vowel/consonant immediately after its onset and squeeze either the precision or the power grip device at the same time with the vocalization. In line with the proposal of Ramachandran and Hubbard (2001), the paradigm shows that vocal and manual responses are performed particularly rapidly when there is a hypothesized sound-symbolic match between the vowel/consonant and action (see Figure 1). That is, when the manual response is performed with a precision grip, in comparison to a power grip, and the pronounced vowel is [i] as opposed to [ɑ]. Vocal and manual responses are similarly facilitated when the consonants [t] (an apical stop consonant) and [r] (an alveolar trill) are pronounced with the precision grip response as compared to the power grip response. Tiainen et al. (2017b) showed that although *the sound-grip effect* was originally observed in speakers of Finnish (i.e., a Finno-Ugric/Uralic language), the same effect can be also observed in speakers of Czech (i.e., a Slavic/Indo-European language), which belongs to a different language family than Finnish.

Complying with the sound-symbolic view of Ramachandran and Hubbard (2001), *the sound-grip effect* associates the precision grip with the front-close vowel [i] because it is produced by a “small” mouth shape – analogous to the shape of the precision grip – so that the tongue blade is pushed into a high-anterior position. Along the same lines, the precision grip might be associated with the consonants [t] and [r] because articulation of these consonants is achieved by producing a precise closure between the tip of the tongue and the alveolar ridge, which in a sense mimics a finger closure of the precision grip.

**Figure 2:**
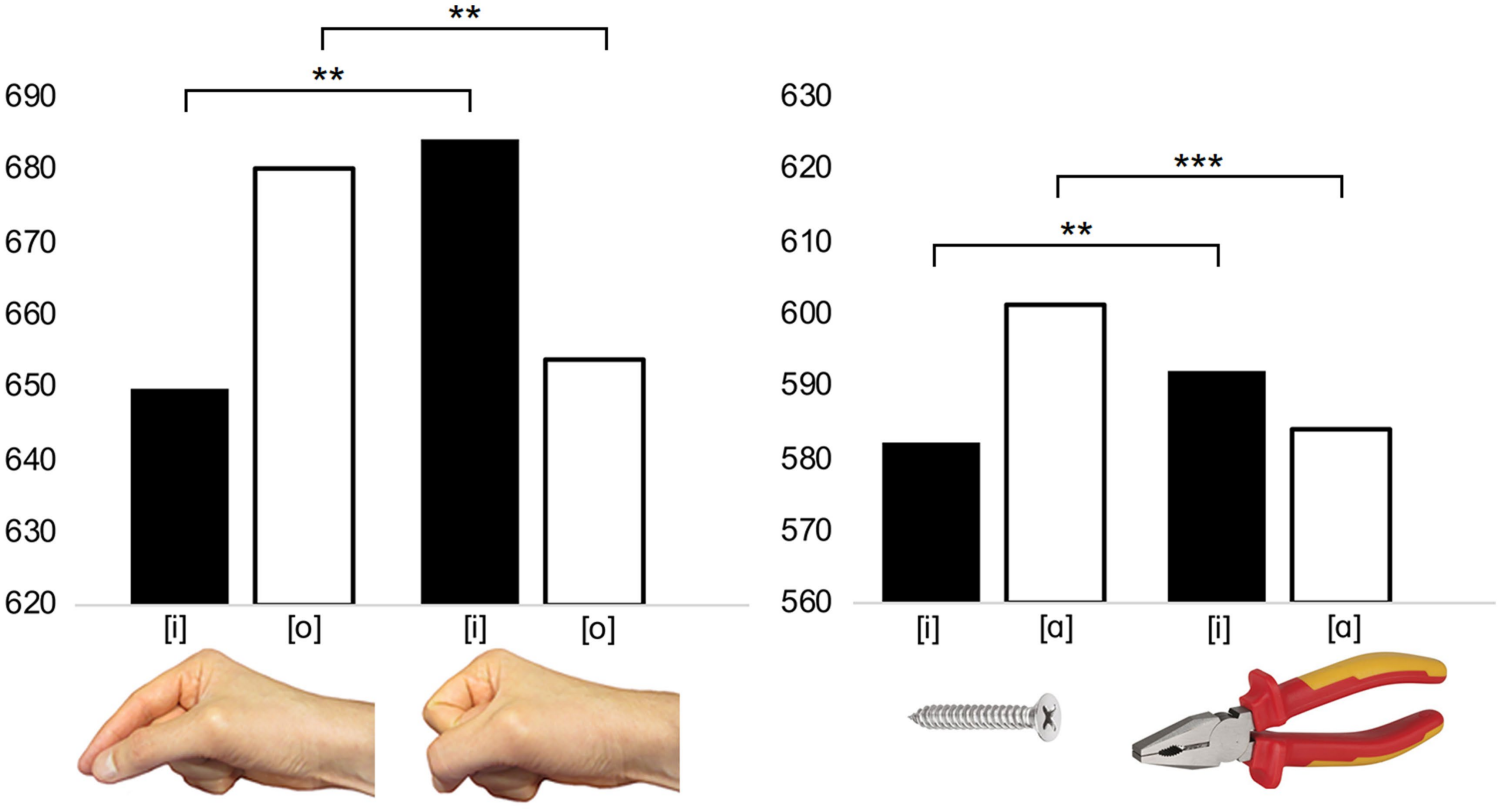
**(Left)** The RT effect for the study published by Vainio et al. (2017a). In the experimental task, the participants were presented with an image of a hand-shaped into the precision or power grip. The participants had to respond, as quickly as possible, by vocalizing the [i] or [o] according to the perspective (above/front) of a hand. [i] responses were produced particularly rapidly when the hand was shaped into the precision grip, and [o] was produced particularly rapidly when the hand was shaped into the power grip. **(Right)** The RT effect for study published by Vainio et al. (2019a). In the experimental task, the participants were presented with an image of an object whose size was compatible with the precision or power grip. The participants had to respond by vocalizing [i] or [a] according to the category of the object (manufactured/natural). [i] responses were produced particularly rapidly when the object’s size was compatible with the precision grip, while [a] responses were produced particularly rapidly when the size was compatible with the power grip. Asterisks indicate statistically significant differences (^***^ *p*<0.001; ^**^ *p*<0.01).

Further studies have shown that the connection between the grip type and a specific phoneme or speech sound is not only observed at the level of action production related to a particular articulatory gesture and grip type, but this connection can also manifest itself at the levels of perceptual and conceptual processing. First, vocalization of [ti] and [i] is facilitated solely by preparing to respond with the precision grip, in comparison to the power grip, in absence of an actual response execution (Tiainen et al., 2017a). Second, when participants are presented aurally with the syllables [ti] and [kɑ] by hampering their discrimination using a noise-mask, a simultaneous grip performance systematically modulates categorization of these syllables: the likelihood of categorizing the syllable as [ti], in comparison to [kɑ], is significantly increased during precision grip performance (Tiainen et al., 2016). Third, hearing the syllable [ti], in comparison to [ka], facilitates precision grip responses as compared to power grip responses (Vainio et al., 2014). Fourth (see Figure 2), the pronunciation of the vowel [i], in comparison to [ɑ], is facilitated by observing an image of a hand that is shaped into the precision grip closure as compared to the power grip closure (Vainio et al., 2017a). Fifth, the pronunciation of the vowel [i], in comparison to [ɑ], is facilitated by an image of an object that is graspable by the precision grip (e.g., a pin) as compared to an object graspable with the power grip (e.g., a bottle; Vainio et al., 2019a). As such, *the sound-grip effect* can be assumed to present a sound-action version of sound symbolism in which a particular vocal sign is iconically associated with a motor, perceptual and conceptual representation of a particular hand action.

The sound-action association, which is similar to that of *the sound-grip effect*, can also be observed in the shaping of the precision grip during the reaching-to-grasp action. Gentilucci and Campione (2011) asked their participants to reach and grasp an object using the precision grasp. The participants executed the action while they pronounced either [i] or [ɑ]. It was found that production of [ɑ], in comparison to [i], significantly increased finger opening during the grasp action. This outcome systematically associates a specific property of a manual action with a particular vocal sign, hence providing evidence for a sound-action interpretation of sound symbolism. Extending this finding to the context of sound symbolism in the light of mouth-gesture accounts (e.g., Paget, 1944; Ramachandran and Hubbard, 2001), the vowel [ɑ] might be associated with the concept of *largeness* partially because, analogously to the increased mouth opening associated with producing the vowel [ɑ], grasping large objects requires relatively large openings between the fingers and the thumb.

Research shows that, in addition to systematically linking particular speech sounds to grasping, sounds can be also associated with reach-related directional hand movements. Originally, Wallace (1881) proposed that in many languages, the lip protrusion (i.e., a roundedness of a vowel) is involved in producing words such as *go* as if a manual pointing gesture was to be replaced by a lip-pointing gesture in articulation. Ramachandran and Hubbard (2001) similarly suggested that, in many languages, articulating the word *you* involves mouth configuration in which lips are protruded as if the articulatory gesture would imitate manual pointing that is directing outward. The view that emphasizes gestural connectivity between lip and manual pointing is in line with a tendency – observed in many cultures – to accompany, or entirely replace, manual pointing gestures with lip and/or head pointing (Enfield, 2001). However, the cross-linguistic study reported by Wichmann et al. (2010) showed that the front vowel [i] is relatively frequently included in the pronoun that points to the hearer, while the back vowel [ɑ] is more frequently included in the pronoun that points to the speaker himself. If the Wallacian mimicry hypothesis is applied to this finding, it appears that manual pointing could be mirrored in articulating particular deictic words so that the articulatory pointing is produced by pushing (i.e., producing front-close vowels) or pulling (i.e., producing back-open vowels) the tongue.

**Figure 3:**
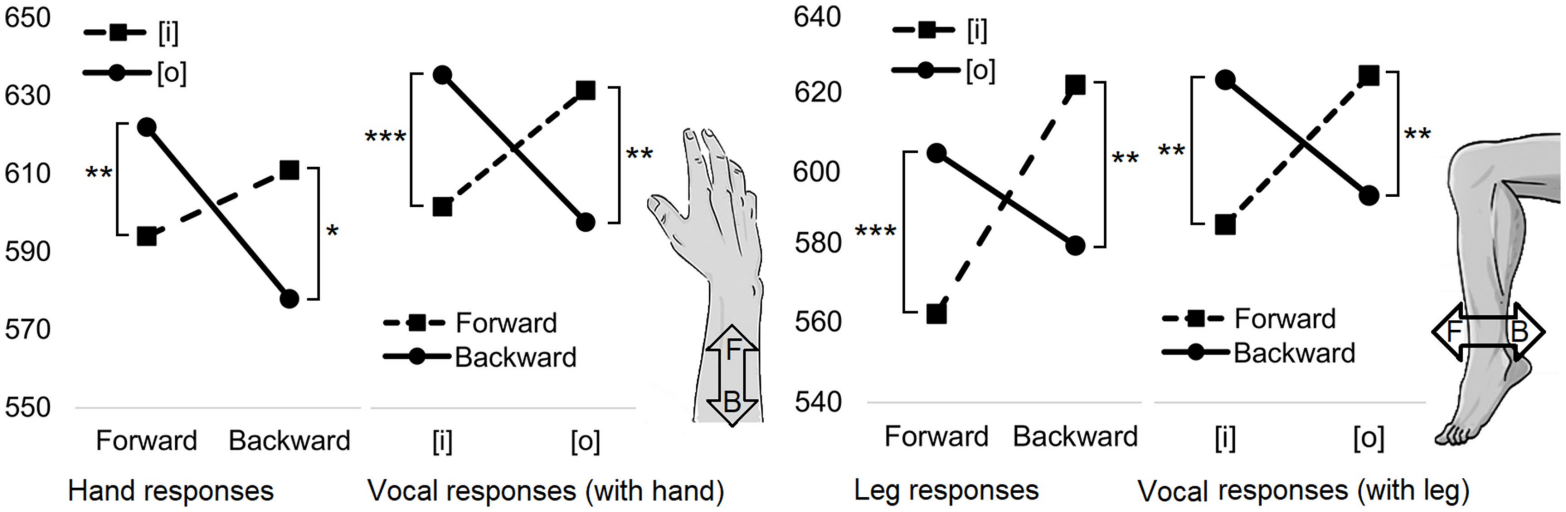
The RT effect for the study published by Vainio et al. (2019b). In the experimental task, the participants were visually presented with the vowel [i] or [o]. The participants had to respond by moving their right hand (the left line graphs) or right leg (the right line graphs) either forward or backward according to the color (green/blue) of the letter while simultaneously pronouncing the vowel. Limb movements and vocalizations were produced particularly rapidly when the limb was moved forward and the vowel was [i], and when the limb was moved backward and the vowel was [o]. In the images of hand and leg, the letter F (inside the arrow) refers to the word “forward” and the letter B refers to the word “backward.” Asterisks indicate statistically significant differences (^***^ *p*<0.001; ^**^ *p*<0.01; and ^*^ *p*<0.05).

Our research has provided experimental evidence for the view discussed above that vowel fronting is associated with outward-directed (i.e., away from the body) hand movement, while vowel backing is associated with inward-directed (i.e., toward the body) hand movement (Vainio et al., 2015, 2018). The study used a dual-action paradigm that was a modified version of *the sound-grip effect* task. In the task, participants were presented visually with a vowel in green or blue, and they were required to perform a push or pull movement with a joystick according to the color, while simultaneously pronouncing the vowel presented. In this *sound-reach effect* (see Figure 3 for the effect), the vocal and manual responses were facilitated when there was a match between an articulatory fronting/backing property of the vowel and the direction of hand movement. These response benefits were observed when the outward directed hand movements were performed with the vowels that required relative vowel fronting such as [ø] (a rounded front-mid vowel), and when the inward directed hand movements were performed with the vowels that required relative vowel backing such as [o] (a rounded back-mid vowel). In addition, like *the sound-grip effect*, this *sound-reach effect* can be found in Finnish and Czech speakers (Tiainen et al., 2017b). Moreover, more recent research has revealed that the same effect can be observed when the manual inward-outward responses are replaced with corresponding leg responses (Vainio et al., 2019b). This suggests that the effect is not restricted to manual processes, but rather the effect can manifest itself in relation to inward-outward movements produced by any effector of the body. Hence, one might propose that the effect essentially operates within inward-outward concepts that are grounded in body movements (Vainio, 2019). Thus, this sound-reach effect presents a sound-action version of sound symbolism in which a particular vocal sign is iconically associated with the direction of a body movement. Below, we describe the sensorimotor processes that might underlie sound symbolism phenomena (i.e., sound-action symbolism) that associate a specific speech sound with an action concept.

## Embodiment of Concepts

Concepts are traditionally defined as abstract mental entities, different from the motor or perceptual processes (Quillian, 1968; Machery, 2007). This view holds that symbolic cognition is achieved by transforming sensory and motor information into a common amodal representation format (Pylyshyn, 1985). In line with these views, for instance, Mahon and Caramazza (2008) have proposed that all concepts are represented at an abstract, a modal level and motor activation, which is often observed with processing conceptual information, is a by-product of activation spreading from task-related perceptual and/or conceptual processing.

In a sharp contrast, the view that the meaning of certain non-arbitrary vocal signs is grounded in motor processes is consistent with theories of grounded (or embodied) cognition. These theories assume that concepts are represented in the neural networks of distinct brain areas responsible for modality-specific processes of sensory, motor, and emotional systems (Barsalou, 2008; Pulvermüller and Fadiga, 2010). Similarly to the embodied models of cognition, hybrid models also assume that motor and perceptual systems contribute to conceptual representations (Binder and Desai, 2011). However, differently from the accounts of “strong embodiment” (Meteyard et al., 2012), the hybrid models assume that representing conceptual knowledge involves the functioning of semantic hubs that serve to bind distinct modality-specific processes (Kiefer and Pulvermüller, 2012). Although, increasing amount of evidence supports the hybrid models, the debate concerning the nature of conceptual representation is still open (Mahon and Hickok, 2016).

Hybrid and grounded cognition accounts generally hold that motor processes play a key role in representing many concepts; most obviously, those concepts that are associated with actions (Barsalou, 2008). In line with this, studies have shown, for example, that verbs (Hauk et al., 2004) and objects (Creem-Regehr and Lee, 2005) that are associated with body movements (e.g., the word “*grasp*”) are represented in the motor representations of the body parts that are involved in performing these actions. For example, transcranial magnetic stimulation (TMS) research has found corticospinal facilitation associated with hand muscles during passive observation of graspable objects (Franca et al., 2012). Additionally, TMS applied to hand and leg motor representations influence semantic processing of hand and leg related action-words (e.g., “pick” vs. “kick”), respectively, providing causal evidence for embodied views (Pulvermüller et al., 2005).

Some research has suggested that the motor system is fundamentally involved in representing even abstract linguistic concepts such as emotions. According to Pulvermüller (2018), abstract emotional concepts are grounded in concrete actions, such as emotion-related facial expressions, head movements, hand gestures, and body postures. Thus, the meaning of *anger* could be grounded in action representations of, for example, angry facial expressions, tight postures, and closed fists. This view assumes that grounding the meaning of an abstract emotional concept in concrete actions enables the communication and labeling of inner states that are not accessible as such to other individuals in a robust, shareable, and concrete manner. Even numerical concepts (e.g., “9”) have been proposed to be partially represented in relation to actions (Tschentscher et al., 2012; Vainio et al., 2019c). For example, behavioral studies have shown that perceiving relatively small numbers (e.g., 1–4) facilitate responding with the precision grip, while larger numbers (e.g., 6–9) facilitate responding with the power grip (Lindemann et al., 2007; Moretto and Di Pellegrino, 2008). Similarly, when participants grasp an object on which a small or large number is printed, the grasp aperture is increased when the number is large as compared to small numbers (Andres et al., 2004).

The findings related to embodiment of number magnitudes are in line with the A Theory of Magnitude (ATOM) theory (Walsh, 2003), which assumes an overlap in sensorimotor processes that represent conceptual magnitude for the metrics of time, space, and quantity. This account proposes that the fronto-parietal sensorimotor network that is largely responsible for transmitting perceptual information of spatial and temporal metrics to action planning processes (Milner and Goodale, 2008) – planning manual actions, such as grasping, reaching, and pointing in particular – is closely involved in representing magnitude concepts (e.g., small number, near distance, short duration, and small size) in generalized and abstracted form. That is, for instance, the same sensorimotor processes that enable preparing of the precision grip according to the small size of an object also enable the representation of a concept of *smallness* relative to magnitude dimensions of size, duration, length, and so forth (Bueti and Walsh, 2009).

### How Sound Symbolism Could Be Grounded in Motor Processes?

If concrete action-related concepts as well as loosely action-related abstract concepts (e.g., numbers, magnitudes, and emotional concepts) are indeed grounded in actions, one might assume that many sound symbolism phenomena – at least those that are somehow related to actions – are similarly grounded in motor processes. In this light, what makes the ATOM theory potentially relevant in the context of sound-magnitude symbolism is the way in which it assumes that a particular magnitude concept (e.g., *smallness*) is grounded in the same action representation irrespective of magnitude type (e.g., size, length, and duration). Sound symbolism research has similarly shown that the concept of *smallness* is associated with the same vowels irrespective of the magnitude dimension. Front-close vowels are sound symbolically associated with quick movement, small size, short spatial, and temporal length as well as near distance, while back-open vowels are associated with slow movement, large size, long spatial, and temporal length as well as far distance (Sapir, 1929; Tanz, 1971; Cuskley, 2013; Rabaglia et al., 2016; Bross, 2018; Vainio, 2021). In addition, just as the ATOM theory grounds generalized magnitude representations in manual actions, in the context of sound symbolism, small magnitudes are linked not only to front-close vowels, but also to precision grasping (Vainio et al., 2013, 2017a, 2019a). Thus, given that mouth gestures and manual gestures are programmed within a combined sensorimotor network (e.g., Arbib, 2005; Gentilucci and Corballis, 2006; Vainio, 2019), it could be assumed – in the light of the ATOM theory – that this mouth-hand network is involved in representing the concept of magnitude in abstracted and generalized from. Therefore, *the sound-magnitude effect* that associate particular vowels (e.g., [i]) with a particular magnitude (e.g., *smallness*), might be at least partially based on grounding this magnitude concept in a shared network representing the precision grasp (Vainio et al., 2013), a closed grasp aperture (Gentilucci and Campione, 2011), and a front-close articulatory gesture (Vainio, 2021).

In the nutshell, the evidence for the view that the sensorimotor mouth-hand network is involved in representing the concept of magnitude, and that this sensorimotor grounding of magnitude might provide the neural basis for *the sound-magnitude symbolism*, is 3-fold. First, it has been shown that seeing an object that is graspable with the precision grip facilitates precision grip responses, while power grip-compatible objects facilitate power grip responses (Tucker and Ellis, 2001; Ellis et al., 2007). Indeed, an object’s size-grasp affordance, signaling how an object could be optimally grasped, is implicitly represented for a viewed object within the parieto-frontal network responsible for planning visually-driven actions (Grèzes et al., 2003; Kourtis et al., 2018), and automatically activates a grasp motor program that is compatible with the object’s size (Franca et al., 2012; Makris et al., 2013). Second, evidence suggest a systematic interaction between specific speech sounds and grasping (e.g., Arbib, 2005; Gentilucci and Corballis, 2006; Vainio, 2019). As already mentioned, for instance, the precision grasping is associated with front-close vowels and apical consonants (Vainio et al., 2013, 2018). Third, perceptual and conceptual processing of an object’s size-grasp affordances not only recruits grasp representations (Tucker and Ellis, 2001; Franca et al., 2012) but also appears to recruit vowel production processes (Vainio et al., 2019a). Taking this evidence together, it appears that mechanisms that transform size-grasp affordances into corresponding grasp- and articulation-related motor programs might provide a neural basis for sound-magnitude symbolism phenomena.

Regarding *the sound-reach effect* (Vainio et al., 2015), if the embodied account is applied to explain the effect in the light of the ATOM theory, it would be tempting to propose that the front vowels are associated with outward-directed body movements and the back vowels with inward-directed body movements because spatial knowledge of direction is partially represented in the context of body movements that, in particular, integrate directional tongue and hand movements. Hence, it could be concluded that, for example, discrete but semantically overlapping concepts, such as “*outward*,” “*forward*,” and “*away from the body*” are conceptually represented and generalized within a motor network that programs forward-directed movements of the tongue and the hand in a relatively integrated manner. Consequently, the sound symbolism effect that connects, for example, front vowels to the concept of *outward*, is to some extent based on the grounding of this concept in action representations of forward-directed body movements performed particularly with the hand and tongue.

Finally, we propose that the sound-action symbolism effects (e.g., Vainio et al., 2013, 2015), in particular, are based on a motor network that connects articulatory gestures to the iconically analogous actions of other body parts, in particular hand actions. This view assumes that these sound-action symbolism effects arise from the grounding meaning of actions in this combined motor network, consequently associating a concept with a specific articulatory gesture (i.e., vowel and/or consonant). In general, following the embodied accounts of conceptual representation, semantic concepts that are associated with actions and body movement (e.g., prehensile hand movements, object affordances, and emotions *via* body expressions) are grounded to some degree in motor representations. Therefore, it is likely that sound symbolism phenomena that refer to these same concepts are also grounded in motor processes. This is not to say that this motor grounding hypothesis can be applied to sound symbolism effects that are not related to body movements. This section and the “Sound-Action Symbolism” provide some evidence for supporting this view. The next section provides linkages between sound symbolism, prosody, and body gestures. Essentially, the next section emphasizes that similarly to some sound symbolism phenomena, prosody, and communicative body gestures also signal meaning iconically and in an embodied manner.

## Suprasegmental Sound Symbolism: Associating Prosody with Sound Symbolism and Body Movements

Excepting onomatopoeic words, prosody (i.e., the suprasegmental speech features consisting of voice fundamental frequency, voice intensity and quality, as well as the rhythmic aspects of speech) provides perhaps the most explicit example of iconical conveyance of meaning through the sound properties of oral signaling. For example, prosodic emphasis on a word, produced by increasing its loudness, pitch, and duration, is used to iconically highlight the magnitude of a concept (e.g.,” *it is SOO cold*”). Indeed, empirical evidence shows that English speakers are able to relatively accurately categorize Japanese words when the words are produced with expressive prosody (Kunihira, 1971). Prosody can also cue semantic distinctions like cold-hot or strong-weak (Nygaard et al., 2009; Reinisch et al., 2013). Finally, relevantly for the current proposal, it has been shown that prosody contributes to the effects of sound symbolism (Dingemanse et al., 2016), and prosodic speech events are temporally synchronized to the production of ideophones (Kita, 1997). This evidence show that prosody can iconically convey the meaning of a referent as segmental sound symbolic vocal signs do, and that expressing prosody is fundamentally coupled with expressing sound symbolic speech elements.

Speech is frequently accompanied by gestures (McNeill, 2012). These co-speech gestures, produced particularly by the hands, head, and face, provide a communicative repertoire that can be used to communicate or emphasize the meaning of a referent. Importantly for the current proposal, expressing prosody and sound symbolism are tightly grounded in gestural body movement. Gesturing with the head, eyebrows, and by using beat gestures (i.e., simple and fast movements of the hands) are often observed in relation to the production of suprasegmental speech features, such as stress, intonation, rate, and rhythm (Wagner et al., 2014). For example, the intonation peak, which is observed in question intonation or when providing a prosodic stress on a word, has been shown to frequently co-occur spontaneously in synchrony with gestural hand (Esteve-Gibert and Prieto, 2013; Krivokapic et al., 2015), head (Graf et al., 2002; Esteve Gibert et al., 2014), and eyebrow (Flecha-García, 2010; Swerts and Krahmer, 2010) movements. In addition, prosody that signals an affective state (i.e., emotional prosody) is typically encoded and decoded, in a systematic manner, in relation to emotional facial gestures (Hietanen et al., 1998; Russell et al., 2003) and body postures (Stienen et al., 2011). All this evidence shows that like sound symbolism, prosody is also consistently associated with gestural motor processes.

Research has also shown that there is a great universal tendency in the sound-meaning mapping of prosodic cues. It has been proposed that about 70% of typologically dissimilar languages have a tendency to use a rising pitch to provide a prosodic cue about interrogation as opposed to affirmation (Bolinger, 1978). As another example, regarding non-linguistic prosody, joy is typically expressed in speech by increased pitch of vocal signaling across different languages, while sadness is expressed by lowering the pitch (Chung, 1999). The universal tendency concerning these phenomena suggests that these sound-meaning associations are not based on convention. As a consequence, the question arises: why a particular prosodic feature (e.g., a rising pitch) signals, across cultures, a particular meaning (e.g., question/joy)?

If viewed through embodied accounts of cognition, signaling a particular meaning, for example, with rising pitch could be somehow grounded in motor processes. Indeed, research shows that a rising pitch in question intonation is associated with spontaneous rising of head and eyebrows (McClave, 1991; Horstmann and Ansorge, 2011). There can be several reasons why question is communicated by head rising. For example, head rising might highlight the emotional surprise content of signaling (Bolinger, 1983), or the head rising gesture might develop, in the context of communicating a question, from a request to be picked up by an infant with raised arms and head (Rossano, 2013). However, although these speculations might explain why a question is communicated by the head nod, they do not explain why a question is communicated by a rising pitch in addition to the head nod. It has been proposed that rising pitch in intonation is grounded in the body so that moving the head upward changes the position of the larynx, which pulls on the cricothyroid muscle and consequently changes the pitch (Cwiek and Fuchs, 2019). Hence, the pitch rise in question intonation can be ultimately a consequence of a particular body movement, which gesturally signals question, and has consequently become a universal prosodic standard when signaling a question. This logic could be similarly applied to a rising pitch associated with expressing joy, given that joy is typically expressed by a particular body posture in which head is tilted upwards (Dael et al., 2012). As such, we propose that the meaning of a particular prosodic feature, such as a pitch rise, is essentially grounded in gestural body movements. Intonation and emotional prosody can thus be seen as auditory gestures that iconically simulate body gestures using the vocal apparatus. Taken together, the views and evidence discussed above hold that prosody largely originates from the same representational embodied processes as the sound-action symbolism and communicative body gestures that enable a vivid and iconic expression of the meaning of a referent to the recipient.

## Conclusion

Evidence shows that there exists a category of sound symbolism effects – largely neglected in the literature of sound symbolism – that associates a vocal sound with a particular body action. This sound-action symbolism appears to operate within the levels of motor, perceptual, and conceptual representations. This is supported, for instance, by the fact that *the sound-grip effect* – the aforementioned example of sound-action symbolism − is observed relative to the action execution, perception, and conceptualization. Emphasizing the mouth-gesture hypothesis in theorizing these effects, they may essentially be grounded in neural interaction between action representations of mouth and other body parts, the hands in particular. That is, the sound-action symbolism effects might arise from grounding the meaning of actions in the motor network combining articulatory representations with action representations of other body parts, consequently associating a concept with a specific articulatory gesture (i.e., vowel and/or consonant). These views of sound-action phenomena are in line with embodied views of cognition, according to which iconicity provides the mechanism for the grounding of language in sensorimotor systems (Perniss and Vigliocco, 2014).

Moreover, we propose that not only are sound-action symbolism phenomena based on these embodiment mechanisms, but also that some sound symbolism effects that have not been traditionally explained in terms of embodied accounts of cognition (e.g., *the sound-magnitude symbolism*) can be also based on the grounding of conceptual representations in sensorimotor processes. Furthermore, in the light of the motor chauvinist perspective, the paper emphasizes that sound symbolism, prosody, and body gestures might have a common origin in expressing ideas using utterances and body movements in order to communicate a meaning of a referent in an iconic manner.

Finally, for sake of verifying the view that the *sound-grip effect* is indeed based on the same sound symbolism processes as the *sound-magnitude effect*, future studies should investigate the *sound-grip effect* in a systematic manner in order to show that the same consonants that are associated with small/large objects in the sound-magnitude symbolism are also associated with the precision/power grip responses, respectively. Moreover, although the sound-action symbolism effects introduced in this review present clear examples of sound-meaning iconicity, they request for future cross-linguistic research on whether these sound-action phenomena have established themselves in the lexicons of spoken languages. As an example, it should be investigated whether words that refer to precision grip-related concepts contain more frequently front-close vowels than words that refer to power grip-related concepts. In addition, future research should explore whether motor processes of mouth and/or hand provide causal effect on sound symbolic mapping of small/large sizes with specific vowels, for example, by temporarily disrupting hand and/or mouth motor processes using repetitive Transcranial Magnetic Stimulation (rTMS), while participants carry out a sound symbolism tasks. Finally, the proposal that prosodic cues could be also grounded in motor processes request for future research.

## Author Contributions

LV and MV played a central role in writing this manuscript and contributed equally to the new perspectives of the manuscript. All authors contributed to the article and approved the submitted version.

## Conflict of Interest

The authors declare that the research was conducted in the absence of any commercial or financial relationships that could be construed as a potential conflict of interest.

## Publisher’s Note

All claims expressed in this article are solely those of the authors and do not necessarily represent those of their affiliated organizations, or those of the publisher, the editors and the reviewers. Any product that may be evaluated in this article, or claim that may be made by its manufacturer, is not guaranteed or endorsed by the publisher.
